# The Tetrel Bond and Tetrel Halide Perovskite Semiconductors

**DOI:** 10.3390/ijms24076659

**Published:** 2023-04-03

**Authors:** Pradeep R. Varadwaj, Arpita Varadwaj, Helder M. Marques, Koichi Yamashita

**Affiliations:** 1 Department of Chemical System Engineering, School of Engineering, The University of Tokyo, 7-3-1, Tokyo 113-8656, Japan; 2 Molecular Sciences Institute, School of Chemistry, University of the Witwatersrand, Johannesburg 2050, South Africa

**Keywords:** tetrel halide perovskites, DFT calculations, MESP and QTAIM analyses, geometries and energetics, tetrel bond, alkali bond, ion-pair chemistry

## Abstract

The ion pairs [Cs^+^•TtX_3_^−^] (Tt = Pb, Sn, Ge; X = I, Br, Cl) are the building blocks of all-inorganic cesium tetrel halide perovskites in 3D, CsTtX_3_, that are widely regarded as blockbuster materials for optoelectronic applications such as in solar cells. The 3D structures consist of an anionic inorganic tetrel halide framework stabilized by the cesium cations (Cs^+^). We use computational methods to show that the geometrical connectivity between the inorganic monoanions, [TtX_3_^−^]_∞_, that leads to the formation of the TtX_6_^4−^ octahedra and the 3D inorganic perovskite architecture is the result of the joint effect of polarization and coulombic forces driven by alkali and tetrel bonds. Depending on the nature and temperature phase of these perovskite systems, the Tt···X tetrel bonds are either indistinguishable or somehow distinguishable from Tt–X coordinate bonds. The calculation of the potential on the electrostatic surface of the Tt atom in molecular [Cs^+^•TtX_3_^−^] provides physical insight into why the negative anions [TtX_3_^−^] attract each other when in close proximity, leading to the formation of the CsTtX_3_ tetrel halide perovskites in the solid state. The inter-molecular (and inter-ionic) geometries, binding energies, and charge density-based topological properties of sixteen [Cs^+^•TtX_3_^−^] ion pairs, as well as some selected oligomers [Cs^+^•PbI_3_^−^]*_n_* (*n* = 2, 3, 4), are discussed.

## 1. Introduction

All-inorganic [1,2,3] and organic–inorganic [4,5,6,7] metal halide perovskites are a special class of semiconducting materials used in photovoltaics [8], photodetectors [9], and photocatalysis [10,11]. The continuing development of a variety of these materials has been well documented [12,13,14]. The hybrid organic–inorganic metal halide perovskites have appeared in a variety of dimensions, *n*D (*n* = 0–3) [15,16,17,18], as have the all-inorganic tetrel halide perovskites [19,20,21].

The well-known 3D organic–inorganic metal halide perovskites have the generic formula ATtX_3_ (A = MA^+^, methylammonium [CH_3_NH_3_^+^]; FA^+^, formamidinium [HC(NH_2_)_2_^+^]; Tt = Pb, Sn, Ge; X = Cl, Br, I). The all-inorganic counterparts are obtained when the A-site cation is replaced by an inorganic moiety, such as Cs^+^, Rb^+^, or K^+^, for example. The first type has been somewhat misleadingly referred to as organo-metal halide perovskites [8,9,17,22]; they are now referred to as single metal halide perovskites [23,24], with generic formula ABX_3_, where A is a monovalent cation, B is a divalent metal cation, and X is a monovalent halide anion [25]. ATtX_3_ comprises an anion (TtX_3_^−^) and a cation A^+^, with Tt formally in its +2-oxidation state.

Three-dimensional metal halide perovskites form cage-like structures [15,16], while 2D [26], 1D [17], and 0D [17,27] perovskites form layered-, chain-, and quantum dot-like structures, respectively. At first, positively charged inorganic or organic moieties (e.g., Cs^+^ or MA^+^) are incorporated inside a negatively charged inorganic architecture. The inorganic skeleton (i.e., [TtX_3_^−^]_∞_) of the crystal in 3D is formed from a negatively charged repeating unit ([TtX_3_^−^]) and these are linked to each other in an infinite array in the presence of the inorganic or organic cations. Why these negatively charged units are repeated in an infinite crystal appears not to have been discussed in the perovskite literature, nor have their bond-specific roles. A number of studies have shown that the extrema of the valence and conduction bands in these 3D materials are driven by orbital contributions arising from the inorganic framework formed by the M and X atoms, and the A-site cation is not involved in contributing to or elevating the functionality of these materials [28,29]. However, other studies have demonstrated that the organic cation plays an important role in determining the hole density of some low-dimensional metal halide perovskites [30]. Indeed, there has been some controversy concerning the actual role played by the A-site cation [31,32,33,34,35].

The aim of this study is to shed light on one of the pressing issues mentioned above. In particular, we shall demonstrate that when a TtX_3_^−^ (X = Cl, Br, I) anion is placed near the electrostatic field of an inorganic cation, such as Cs^+^, they not only like each other by a coulombic attraction, but the electric field of the positively charged moiety Cs^+^ is able to polarize the charge density on the electrostatic surface of TtX_3_^−^. This results in the formation of electron density-deficient (positive) sites on the electrostatic surface of TtX_3_^−^ in [Cs^+^•TtX_3_^−^] (Tt = Pb, Sn, Ge; X = I, Br, Cl, F) ion pairs, except when Tt = Si; these are the building blocks of the 3D structure of CsTtX_3_. The strength of these positive sites on Tt is sufficient to attract nucleophiles on the surfaces of the halogen derivative on another similar interacting unit of TtX_3_^−^, and a repetition of this process results in the formation of an infinite inorganic framework in the presence of Cs^+^ ions. We show that the attractive interaction between a pair of TtX_3_^−^ units in a pair of ion pairs is nothing other than a “tetrel bond” [36]. The physical chemistry of the tetrel bond, and other non-covalent interactions (e.g., the alkali bond [37]), in a variety of [Cs^+^•TtX_3_^−^] ion pairs and in the [Cs^+^•PbI_3_^−^]*_n_* (*n* = 2, 3, 4) oligomers is discussed.

A tetrel bond occurs in chemical systems when there is evidence of a net attractive interaction between an electrophilic region associated with a covalently or coordinately bonded tetrel atom (or atoms) in a molecular entity and a nucleophilic region in another, or the same, molecular entity [36]. In other words, the tetrel bond occurs when an electrophilic σ-hole on the surface of the Tt atom in a molecular entity R–Tt is in coulombic engagement with the nucleophile on the same or another molecular entity. An electrophilic σ-hole is an electron density-deficient region of positive electrostatic potential that appears on the electrostatic surface of an atom A opposite to the R–A covalent bond and has the ability to sustain an attractive bonding engagement with the negative site [38,39,40,41].

This study employs Density Functional Theory (DFT) and focuses primarily on the ion-pair systems of [Cs^+^•PbI_3_^−^]*_n_* (*n* = 1, 2, 3, 4), but Quantum Theory of Atoms in Molecules (QTAIM) and Molecular Electrostatic Surface Potential (MESP) calculations are also performed on [Cs^+^•TtX_3_^−^] (Tt = Si, Ge, Sn X = F, Cl, Br) ion pairs that are supposedly the building blocks in 3D tetrel halide perovskites. The study shows that the formation of the inorganic framework and the geometric stability of cesium tetrel halide perovskite systems arise from the same underlying interactions, tetrel and alkali bonds. Crystalline cesium tetrel halide perovskites were found by searching the Inorganic Crystal Structure Database (ICSD) [42,43] and can also be found on the Materials Project Database [44].

## 2. Results

### 2.1. Illustrative Crystal Geometry of Cesium Tetrel Halide Perovskites

**Figure 1:**
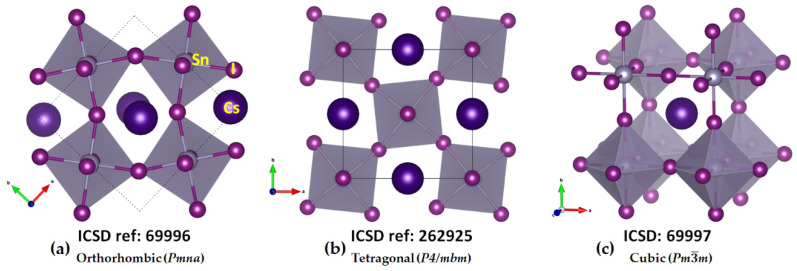
Mixed polyhedral and ball-and-stick models of crystal structures of the semiconductor CsSnI_3_ in the low- and high-temperature phases: (**a**) orthorhombic (*γ*-phase); (**b**) tetragonal (*β*-phase); (**c**) cubic (*α*-phase) [54]. Atom labeling is shown in (**a**). Each polyhedron represents the SnI_6_^4−^ octahedron. The ICSD reference code is shown in each case.

The crystal structures of CsGeX_3_ (X = Cl, Br, I) have been known for some time [45]. The crystal structure of the high-temperature *Pm*$\overset{-}{3}$*m* phase of CsPbI_3_ was reported in 2008 [46], while the low-temperature orthorhombic structures *Pmnb* (ICSD ref: 27979), *Pbnm* (ICSD: 434338; 21955) [47], *Pnam* (ICSD ref: 264725) [48], and *Pnma* (ICSD ref: 32301–32314; 161480; 29350; 27484; 20759; 17016) [49] of the same system have been known since 1959. Regardless of the nature of the temperature phase, the TtX_6_^4−^ framework is common, featuring corner-sharing octahedra. These are tilted in the low-temperature phases along the crystallographic axes but linearly arranged in the high-temperature cubic phase [6,50,51]. These features are evident in the orthorhombic, tetragonal, and cubic phases of CsSnI_3_ (see Figure 1a–c, respectively). They are also reminiscent of cesium lead halide perovskites, CsPbI_3_. The high-to-low temperature phases of CsPbI_3_ [52] are called the *α* (645K), *β* (510K), and *γ* (325K) phases, with space groups *Pm*$\overset{-}{3}$*m*, *P4/mbm*, and *Pbnm*, respectively. A common feature of these systems is that the TtX_3_^−^ anions are linked to each other through tetrel bonds, a feature revealed in this study, and these bonds are responsible for the formation of the 3D inorganic frameworks. All these perovskite systems mentioned above are semiconductors, and several of them have found application in photovoltaics. However, the black orthorhombic form of CsSnI_3_ features p-type metallic behavior with low carrier density, despite an optical band gap of 1.3 eV [53].

*(a)*  *Cesium Tetrel Iodide Perovskites*

**Figure 2:**
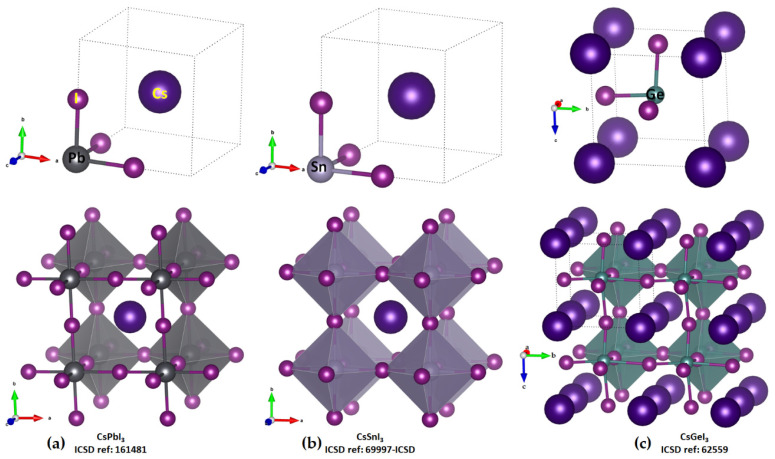
Top: the ball-and-stick models of the unit cells of cesium tetrel iodide perovskites. (**a**) CsPbI_3_; (**b**) CsSnI_3_; (**c**) CsGeI_3_. Bottom: illustration of the polyhedral and ball-and-stick models of corner-shared octahedra for these systems, forming cage-like structures. The ICSD reference is shown in (**a**–**c**).

The unit-cell (top) and cage-like (bottom) structures of cesium tetrel iodide perovskites, CsTtI_3_ (Tt = Pb, Sn and Ge), are shown in Figure 2a–c. The unit cell, an ion pair, [Cs^+^•TtI_3_^−^], when periodically expanded, reveals a cage-like structure. The formation of the latter is driven by Tt···I tetrel bonds, which are equivalent and linear when Tt = Pb and Sn and quasi-linear and non-equivalent when Tt = Ge. For instance, the distances associated with the Tt–I coordinate bonds within the [TtI_3_^−^] fragment and Tt···I tetrel bonds between a [TtI_3_^−^] pair are 3.145 and 3.110 Å in cubic (*Pm-*$\overset{-}{3}$*m)* CsPbI_3_ (Figure 2a) and CsSnI_3_ (Figure 2b), respectively. Because of the small size of the Ge cation, CsGeI_3_ perovskite is not cubic (Figure 2c) and crystalizes in the rhombohedral space group *R3m*. Each of the three equivalent Ge–I coordinate bonds within the [GeI_3_^−^] fragment is 2.747 Å, and each of the three equivalent Ge···I tetrel bonds between four [GeI_3_^−^] fragments linked with each other in the CsGeI_3_ is 3.262 Å. The latter are directional but quasi-linear, ∠I–Ge···I = 169.3°, which is equivalent to the tilting angle, ∠Ge···I–Ge = 169.3°, of the octahedra along each of the three principal axes. There is no structure of CsSiI_3_ deposited in the ICSD.

**Figure 3:**
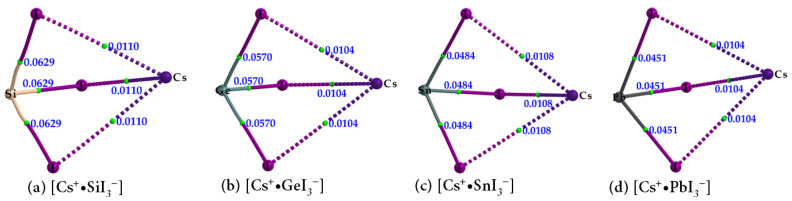
(**a**–**d**) QTAIM-based molecular graphs of cesium tetrel halide perovskite ion pairs, [Cs^+^•TtI_3_^−^] (Tt = Si, Ge, Sn, and Pb), obtained with [*ω*B97XD/def2-TZVPPD]. Values represent the charge density (*ρ*_b_/au) at the Tt–I and Cs···I bond critical points. Atoms, bond paths, and bond critical points are shown as large spheres, solid or dotted lines in atom color, and tiny spheres in green, respectively.

In order to shed some light on the why the entirely negative anions [TtI_3_^−^] attract each other, thus forming tetrel bonds between them (Figure 2, bottom), we performed both QTAIM and MESP analyses. The molecular graphs of cesium tetrel iodide perovskite ion pairs, [Cs^+^•TtI_3_^−^] (Tt = Si, Ge, Sn and Pb), are shown in Figure 3a–d. The topology of the bond paths suggests that Tt–I coordinate bonds, characterized by solid lines, are shorter and stronger than the Cs···I close contacts, described by dotted lines; this is in line with the charge density values at their corresponding bcps, meaning that the Cs···I close contacts are weaker than the Tt–I coordinate bonds. Focusing on CsPbI_3_ as a representative example of this series, each PbI_3_^−^ unit, which is a face of the PbI_6_^4−^ octahedron, is involved in an attractive coulombic interaction with Cs^+^, thereby forming the ion pair [Cs^+^•PbI_3_^−^]. As shown in Figure 2a (bottom), a single cation simultaneously interacts with eight PbI_3_^−^ faces of eight PbI_6_^4−^ octahedra, forming a cage-like structure, with Cs^+^ trapped inside the cage formed by the eight PbI_6_^4−^ octahedra. In other words, the arrangement between the anion and the cation in CsPbI_3_ is such that each face of the corner-shared PbI_6_^4−^ octahedra hosts a Cs^+^ cation (cf. Figure 2a).

The question that immediately arises as to why the cesium tetrel halide perovskites with Tt = Pb, Sn, and Ge have been synthesized, but not with Tt = Si. This is answered below. In short, it is the result of the nucleophilic nature of the electrostatic potential on the surface of Si in molecular [Cs^+^•SiI_3_^−^] that prevents self-assembly of these recurring units.

**Figure 4:**
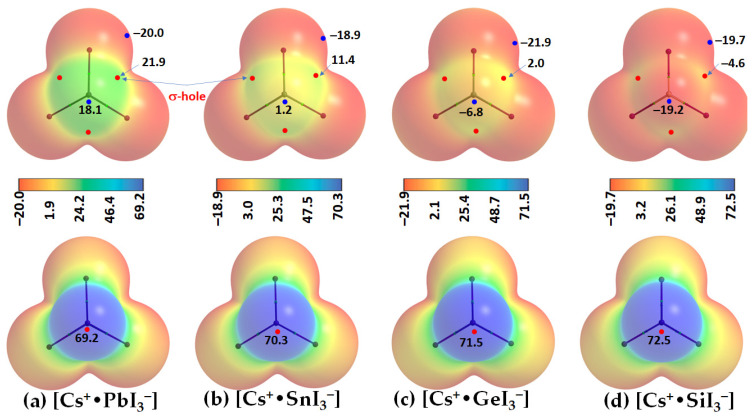
(**a**–**d**) The [*ω*B97Xd/def2-TXVPPD]-level potential on the electrostatic surface of [Cs^+^•TtI_3_^−^] (Tt = Pb, Sn, Ge, Si), mapped on their corresponding 0.001 a.u. (*electrons Bohr^−3^*) isoelectronic density envelopes. The top and bottom panels represent Tt and Cs sites of each CsTtI_3_ facing the reader, respectively. Selected local most maxima and minima of potential (*V_S,max_* and *V_S,min_*) represented by tiny circles in red and blue, respectively, are depicted. Values are given in kcal mol^−1^.

Two distinct features can be readily seen from Figure 4. First, the tetrel atom in the ion pairs carries either a negative or a positive potential (*V_S,max_* < 0 or *V_S,max_* > 0) along the outermost extension of each I–Tt bond. In specific, *V_S,max_* > 0 in [Cs^+^•PbI_3_^−^], [Cs^+^•SnI_3_^−^], and [Cs^+^•GeI_3_^−^], becomes progressively less positive, and switches to *V_S,max_* < 0 in [Cs^+^•SiI_3_^−^]. This is the result of the polarizing field induced by Cs^+^ when it is placed close to the [TtI_3_^−^] anion. The polarization of the surface charge density of Tt in [TtI_3_^−^] is accompanied by an appreciable transfer of charge from the iodide to Cs^+^ cation, varying between 0.085 and 0.116 *e*. This causes the charge density on the surface of Tt to be rearranged in such a manner as to produce depleted regions of charge density on its surface. The charge density-depleted regions appear along the outermost extensions of the I–Tt bonds when Tt = Pb, Sn, or Ge, but not when Tt = Si. These charge density regions on the tetrel atoms along the extension of the three I–Tt bonds are characteristic of σ-holes since they appear opposite to the I–Tt σ coordinate bonds; thus, σ-holes on Tt are electrophilic in [Cs^+^•PbI_3_^−^], [Cs^+^•SnI_3_^−^], and [Cs^+^•GeI_3_^−^] (*V_S,max_* > 0; see Figure 4a–c) but nucleophilic in [Cs^+^•SnI_3_^−^] (*V_S,max_* < 0; Figure 4d).

Secondly, a local most minimum of potential, *V_S,min_*, is found on the Tt atom in each [Cs^+^•TtI_3_^−^], appearing on its surface along the outer extension of the *C_3v_* axis. It is positive in [Cs^+^•PbI_3_^−^] and [Cs^+^•SnI_3_^−^] and negative in [Cs^+^•GeI_3_^−^] and [Cs^+^•SnI_3_^−^]. The strength of the minimum of potential decreases in the order Pb (*V_S,min_* = 18.1 kcal mol^−1^) < Sn (1.2 kcal mol^−1^) < Ge (–6.8 kcal mol^−1^) < Si (–19.2 kcal mol^−1^). This indicates that Pb and Sn are entirely electrophilic, unlike Ge and Si. The MESP graphs also suggest that the stereochemically active lone pair of the Tt sites are squeezed onto the surfaces of the iodides when Tt = Pb and Sn, but not when Tt = Ge and Si. This conclusion is in accordance with a previous study where it was suggested that “a stereochemically active lone pair of electrons of the Pb atom may lie between the two I atoms in the plane” [55]. It was recently argued that the s^2^ lone pair on heavy main-group elements in their lower oxidation states is responsible for the emergence of polar ground states in some ferroic materials and causes a crystallographically hidden, locally distorted state that appears upon warming, a phenomenon referred to as emphanisis [56]. Others have argued that PbO and PbS in both the rocksalt and litharge structures, which have distorted Pb^2+^ octahedra, are not the result of chemically inert, stereochemically active lone pairs, but instead are the result of asymmetric electron densities that rely on direct electronic interaction with the coordinated anions [57]. Further discussion on the importance of stereochemically active lone pairs on Pb in Pb(II) halide compounds can be found elsewhere [58].

The local most maximum potential on the surface of Cs^+^ in the ion pairs is positive, *V_S,max_* > 0, and Cs^+^ becomes increasingly more electrophilic down the series [Cs^+^•PbI_3_^−^], [Cs^+^•SnI_3_^−^], [Cs^+^•GeI_3_^−^], and [Cs^+^•SiI_3_^−^]. This is expected, as Si is relatively more electronegative in the tetrel series, hence, the ability of Cs^+^ to polarize electron density of small-sized Tt decreases. In all cases, both the lateral and axial portions of the halide atoms in [TtI_3_^−^] are entirely negative, with *V_S,min_* < 0, as seen in Figure 4a–d.

Our QTAM results in Figure 3 and Table 1 suggest that the Si–I bonds have appreciable covalency, whereas the Tt–I (Tt = Ge, Sn, and Pb) in [Cs^+^•TtI_3_^−^] have a more ionic character. The Si–I bonds are characterized by negative values of both ∇^2^*ρ*_b_ and *H*_b_ at the Si–I bcps. Although the Tt–I (Tt = Ge, Sn, and Pb) bonds possess ionic character, the negative *H*_b_ values at the Tt–I bcps are indicative of some measure of covalency. These coordinate bonds clearly have mixed bonding character. On the other hand, the cesium-centered charge-assisted alkali bonds, Cs···I, possess closed-shell character (∇^2^*ρ*_b_ > 0 and *H*_b_ > 0) and the charge density at the Cs···I bcps is significantly smaller than that at the Tt–I bcps.

*(b)*  *Cesium Tetrel Bromide Perovskites*

**Figure 5:**
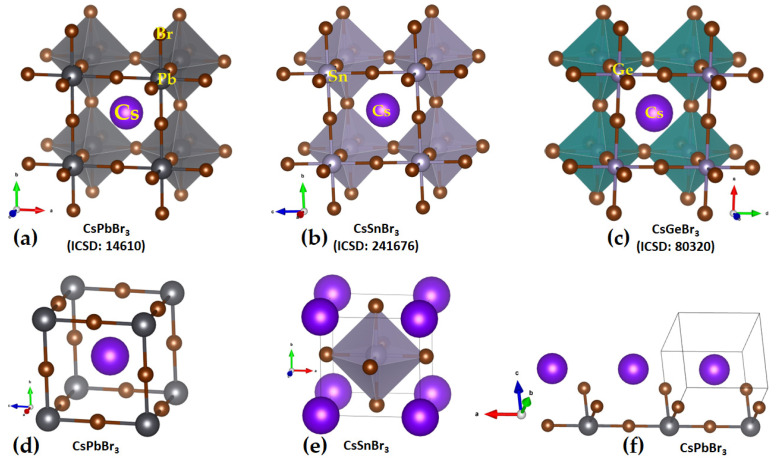
Top: the mixed polyhedral and ball-and-stick models of the cubic (*Pm-*$\overset{-}{3}$*m)* phase crystal structures of cesium tetrel bromide perovskites, showing the corner-sharing octahedra for (**a**) CsPbBr_3_, (**b**) CsSnBr_3_, and (**c**) CsGeBr_3_. Bottom: (**d**) the ball-and-stick model of the cage-like structure of CsPbBr_3_; (**e**) the nature of arrangement of Cs cations on each [SnBr_3_]^−^ face of the SnBr_6_^4−^ octahedra in CsSnBr_3_; (**f**) the 1D array of CsPbBr_3_. The ICSD reference in parentheses is shown in (**a**–**c**).

**Figure 6:**
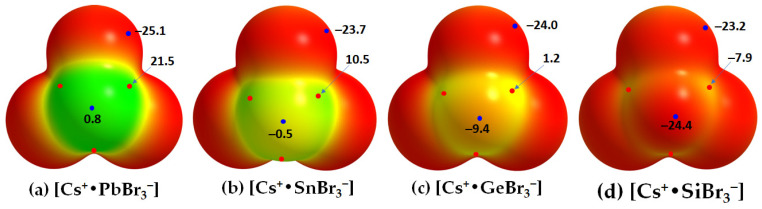
(**a**–**d**) The [*ω*B97Xd/def2-TXVPPD]-calculated potential on the electrostatic surface of [Cs^+^•TtBr_3_^−^] (Tt = Pb, Sn, Ge, Si), mapped on their corresponding 0.001 a.u. isoelectronic density envelopes. The Tt atom in [Cs^+^•TtBr_3_^−^] faces the reader. Selected local most maxima and minima of potential (*V_S,max_* and *V_S,min_*) represented by tiny circles in red and blue, respectively, are depicted. Values are given in kcal mol^−1^. Regions colored blue and red refer to most positive and negative potential, respectively (regions in blue appeared on Cs are not shown).

The cesium tetrel bromide perovskites, [CsTtBr_3_] (Tt = Pb, Sn, Ge), have been reported in different temperature crystalline phases, except for [CsSiBr_3_]; the structures of the high-temperature cubic phase are shown in Figure 5. This is probably because the surface of the Si atom along the Br–Si bond extensions is entirely negative, so the Si atom in the [Cs^+^•SiBr_3_^−^] ion pair is unable to coulombically attract the nucleophilic bromide in a neighboring unit. This is supported by the MESP plots of [Cs^+^•TtBr_3_^−^] shown in Figure 6. They suggest that the surface of Pb in [Cs^+^•PbBr_3_^−^] is entirely positive along and around the outermost extension of the Br–Pb bonds (*V_S,max_* = 21.5 kcal mol^−1^ and *V_S,min_* = 0.8 kcal mol^−1^). That of Sn in [Cs^+^•SnBr_3_^−^] is appreciably positive (*V_S,max_* = 10.5 kcal mol^−1^) along and weakly negative (*V_S,min_* = −0.5 kcal mol^−1^) around the outermost extension of the Br–Sn bonds. In [Cs^+^•GeBr_3_^−^], the surface of Ge is weakly positive along and appreciably negative around the outermost extension of the Br–Ge bonds. However, in the case of [Cs^+^•SiBr_3_^−^], the *V_S,max_* and *V_S,min_* on Si are all negative. For all four ion pairs, both the axial and equatorial portions of the Br atom along and around the Tt–Br extensions are nucleophilic (*V_S,min_* = −25.1 kcal mol^−1^).

**Figure 7:**
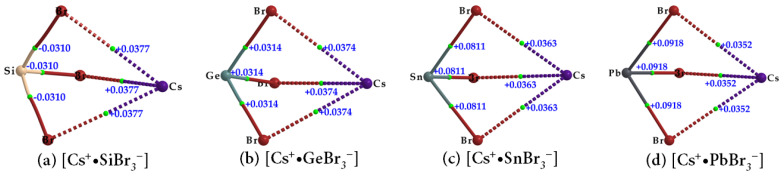
(**a**–**d**) QTAIM-based molecular graphs of tetrel bromide perovskite ion-pairs, [Cs^+^•TtBr_3_^−^] (Tt = Si, Ge, Sn, and Pb), obtained with [*ω*B97XD/def2-TZVPPD]. Values represent the Laplacian of the charge density (∇^2^*ρ*_b_/au) at the Tt–Br and Cs···Br bond critical points. Atoms, bond paths, and bond critical points are shown as large spheres, solid/dotted lines in atom color, and tiny spheres in green, respectively.

The topological charge density characteristics of the Tt–Br and Cs···Br bcps in the ion pairs [Cs^+^•TtBr_3_^−^] (Tt = Si, Ge, Sn, Pb) (Figure 7a–d and Table 2) were found to be very similar to those observed for the Tt–I and Cs···I bcps in [Cs^+^•TtI_3_^−^] (Table 1). However, the charge densities at the Tt–Br and Cs···Br bcps in [Cs^+^•TtBr_3_^−^] were slightly larger; hence the strength of the Tt–Br and Cs···Br bonds are marginally stronger than the Tt–I and Cs···I bonds in [Cs^+^•TtI_3_^−^]. The values of ∇^2^*ρ*_b_ are negative at the Si–Br bcps and positive at the Tt–Br (Tt = Ge, Sn, Pb) bcps, as seen in Figure 7 and listed in Table 2. The extent of charge transfer from the anion to the cation lies between 0.092 and 0.095 *e*.

*(c)*  *Cesium Tetrel Chloride Perovskites*

**Figure 8:**
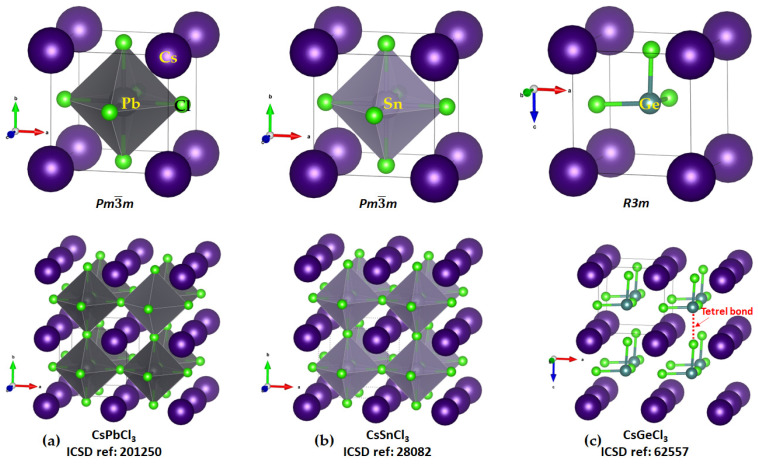
(**a**–**c**) Top: the polyhedral models of the unit cells of the crystals of CsTtCl_3_ (Tt = Ge, Sn, and Pb). Bottom: illustration of the polyhedral models of the 2 × 2 × 2 supercell structures of these crystals, showing their cage-like structures. ICSD references and point groups are depicted in each case. The nature of the tetrel bond is marked in the structure of CsGeCl_3_ (bottom, right).

The solid-state structures of CsTtCl_3_ (Tt = Ge, Sn, and Pb) are known, whereas that of [CsSiCl_3_] has not been reported. The connectivity between the [TtCl_3_^−^] units that lead to the formation of the TtCl_6_^4−^ octahedra in the solid state are evident in all three structures shown in Figure 8a–c. The six Tt–Cl bonds in each polyhedron are equivalent in CsPbCl_3_ and CsSiCl_3_ (2.803 Å and 2.752 Å, respectively), showing that there is very little difference between the three Tt···Cl tetrel and three Tt–Cl coordinate bonds in these systems. In the case of CsGeCl_3_, three of the coordinate bonds are different to the other three; the three Ge–Cl coordinate bond distances are equivalent (2.415 Å each; Figure 8c, bottom) that are appreciably shorter than the three Ge···Cl tetrel bonds (*r*(Ge···Cl) = 3.036 Å and ∠Cl–Ge···Cl = 172.1°).

**Figure 9:**
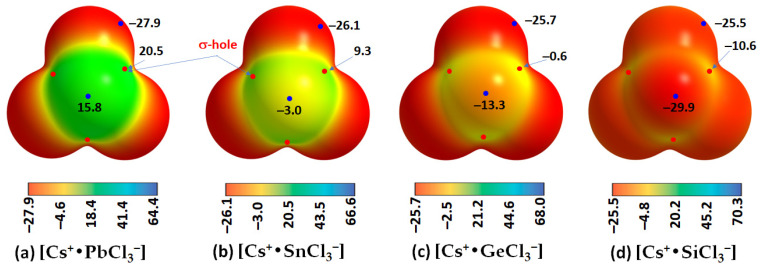
(**a**–**d**) The [*ω*B97Xd/def2-TXVPPD] level potential on the electrostatic surface of [Cs^+^•TtCl_3_^−^] (Tt = Pb, Sn, Ge, Si) ion pairs, mapped on their corresponding 0.001 a.u. isoelectronic density envelopes. The Tt atom in [Cs^+^•TtCl_3_^−^] faces the reader. Selected local most maxima and minima of potential (*V_S,max_* and *V_S,min_*) represented by tiny circles in red and blue, respectively, are depicted. Values are given in kcal mol^−1^.

The fact that tetrel bonding plays a vital role in assembling the [TtCl_3_^−^] units, which leads to the development of the cage-like inorganic framework, is evident from the results of the MESP analysis shown in Figure 9a–c for the ion pairs [Cs^+^•TtCl_3_^−^]. Each of the three Cl–Tt bonds in each ion pair in [Cs^+^•TtCl_3_^−^] (Tt = Pb, Sn) contains electron density-deficient regions along the Cl–Tt bond extensions (σ-holes) with positive electrostatic potentials. However, the potential is weakly negative in [Cs^+^•GeCl_3_^−^] along the three Cl–Ge bond extensions (*V_S,max_* = −0.6 kcal mol^−1^ each, Figure 9c) and strongly negative along the three Cl–Si bond extensions in [Cs^+^•SiCl_3_^−^] (*V_S,max_* = −10.6 kcal mol^−1^). It is clear from these results that the formation of CsTtCl_3_ (Tt = Pb, Sn) perovskite systems in 3D is expected when repeating units of [Cs^+^•TtCl_3_^−^] ion pairs are in close proximity. This kind of assembly is unlikely when [Cs^+^•TtCl_3_^−^] (Tt = Ge and Si) pairs are in close proximity because of coulombic repulsion between the halogen of an ion pair in close proximity to the negative tetrel site in a neighboring unit.

We, and others, have shown on several occasions that caution needs to be exercised when the potential of a σ-hole on an atom in a molecular entity is near neutral. In such a case, a higher isoelectronic density envelope may be required for mapping the potential since the choice of isoelectronic density envelope is arbitrary. Indeed, this is the case with [Cs^+^•GeCl_3_^−^]. When the 0.001 a.u. isoelectronic density was used for mapping, the potential associated with each of the three σ-hole holes on Ge was weakly negative (*V_S,max_* = −0.6 kcal mol^−1^). However, when a 0.0015 a.u. isoelectron density was used, the *V_S,max_* of the σ-holes on the same atom was positive, *V_S,max_* = 4.4 kcal mol^−1^. The positive nature of the σ-hole on Ge explains why Ge in [Cs^+^•GeCl_3_^−^] is capable of attracting the negative portion on the Cl atoms in a neighboring interacting ion-pair, thus leading to the formation of CsGeCl_3_ perovskite crystals in the crystalline phase [45]. By contrast, changing the value of the isodensity envelope did not change the negative character of the σ-holes on the Si atom along the Cl–Si bond extensions; thus, CsSiCl_3_ structures should not be formed when the ion pairs are repeated periodically.

There is a potential maximum on the Cs atom in the ion-pair that appears along the extension of the *C_3v_* axis. Its origin could be due to a weak Tt···Cs interaction in [Cs^+^•TtCl_3_^−^] (Tt = Si, Ge, Sn, Pb), as well as the formation of three equivalent Cs···Cl alkali bonds. The surface of Cs is strongly positive relative to that of Tt in each [Cs^+^•TtCl_3_^−^] (Tt = Si, Ge, Sn, Pb), which rationalizes why the cation lies at the center of the inorganic tetrel halide cage, thus interacting simultaneously with the lone-pair dominant regions of coordinate halides on each of the eight faces of eight octahedra (each sitting at the corner of a cage, Figure 8). 

**Figure 10:**
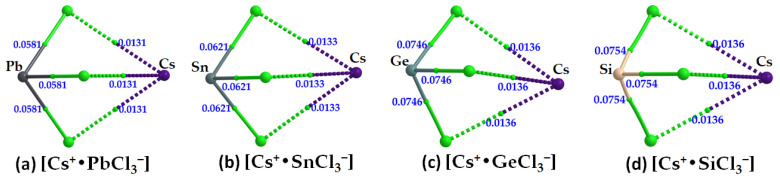
(**a**–**d**) QTAIM-based molecular graphs of cesium tetrel chloride perovskite ion-pairs, [Cs^+^•TtCl_3_^−^] (Tt = Pb, Sn, Ge and Si), obtained with [*ω*B97XD/def2-TZVPPD]. Values represent the charge density (*ρ*_b_/au) at the Tt–Cl and Cs···Cl bond critical points. Atoms, bond paths, and bond critical points are shown as large spheres, solid/dotted lines in atom color, and tiny spheres in green, respectively.

The formation of the alkali bonds in each ion pair, which is expected to mimic what occurs in the crystal (*vide infra*), is evident in the molecular graphs shown in Figure 10a–d and Table 3. The accumulation of charge density at the Cs···Cl bcps is weaker than that at the Tt–Cl bcps. For the latter, it trends as Pb–Cl < Sn–Cl < Ge–Cl < Si–Cl, and, with ∇^2^*ρ*_b_ > 0 and *H*_b_ < 0 (see values in Table 3), these bonds have mixed bonding character. This feature is clearly distinguishable from that of the alkali bonds that are largely electrostatic in character (∇^2^*ρ*_b_ > 0 and *H*_b_ > 0).

*(d)*  *Cesium Tetrel Fluoride Perovskites*

**Figure 11:**
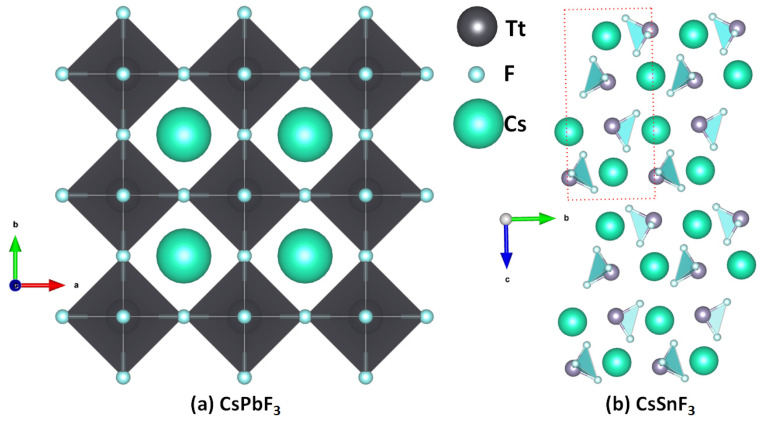
The crystal structure of (**a**) cesium lead fluoride perovskite [CsPbF_3_] (ICSD ref: 93439) and (**b**) low-dimensional cesium tin fluoride [CsSnF_3_] (ICSD ref: 236903).

The structures of [Cs^+^•TtF_3_^−^] are similar to the other cesium halide perovskite ion pairs discussed above. While the formation of these ion-pair systems is likely in the gas phase, they are not all stable in the crystalline phase. The instability of these perovskite structures is arguably due to the mismatch between the cavity of the fluoride-based inorganic perovskite cage formed from the repeating units of [TtF_3_^−^] and the radial size of Cs^+^. This is not the case for CsPbF_3_, as seen in Figure 11a, the structure of which was reported in 1956 (cubic, *Pm*$\overset{-}{3}$*m*, ICSD ref: 30739 [59]) and 2001 (ICSD refs: 93438–93439). Smith et al. [60] have suggested that CsPbF_3_ is the only experimentally synthesized AMF_3_ fluoride perovskite with a polar ground state. Our search of the ICSD showed that CsSnF_3_ is not cubic (space group: P12_1_/n1(14) [61]) and hence is a non-perovskite (Figure 11b). The authors of that study suggested that this system exhibits a ‘zero-dimensional’ crystal structure with isolated SnF_3_^−^ anions separated by Cs^+^ cations; again, this is not surprising since the size of the cage formed by the repeating units of the SnF_3_^−^ anion is too small to accommodate the guest Cs^+^. The ICSD does not contain structures of CsTtF_3_ (Tt = Ge, Sn), but it catalogues crystals such as Cs_2_GeF_6_ and Cs_3_GeF_7_, suggesting that the small size of fluoride, its low polarizability, and its high electronegativity lead it to form other types of crystal structures.

**Figure 12:**
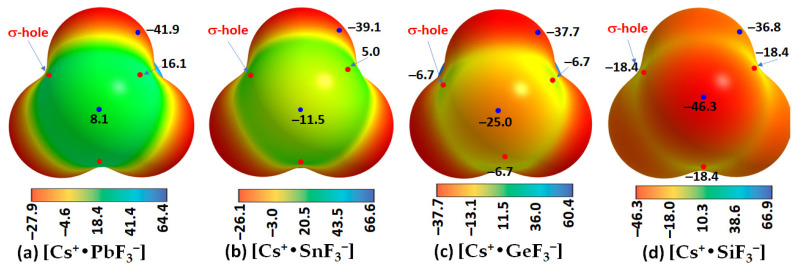
(**a**–**d**) The [*ω*B97Xd/def2-TXVPPD] computed potential on the electrostatic surface of [Cs•TtF_3_] (Tt = Pb, Sn, Ge, Si), mapped on their corresponding 0.001 a.u. isoelectronic density envelopes. The Tt atom in Cs•TtF_3_ faces the reader. Selected local most maxima and minima of potential (*V_S,max_* and *V_S,min_*) represented by tiny circles in red and blue, respectively, are depicted. Values are given in kcal mol^−1^.

The results of our MESP calculations, shown in Figure 12, are in accordance with these rationalizations. They suggest the feasibility of the formation of [CsTtF_3_] (Tt = Pb and Sn) structures in the solid state since the surfaces of the Tt site in the [Cs^+^•TtF_3_^−^] ion-pair systems are highly electrophilic, with the former more so than the latter. Specifically, the surface of Pb in [Cs^+^•PbF_3_^−^] is entirely positive along and around the F–Pb bond extensions (Figure 12a), whereas that of Sn is positive only along the F–Sn bond extensions, while the region around the outer extension of the *C_3v_* axis is highly nucleophilic (Figure 12b). These positive sites are able to engage in a coulombic attraction with the negative site on the halogen of a neighboring unit to form structures of the types shown in Figure 11a,b, respectively. This is not the case when Tt = Ge and Si, the surfaces of which are entirely negative in [Cs^+^•GeF_3_^−^] and [Cs^+^•SiF_3_^−^] (see Figure 12c,d), respectively.

**Figure 13:**
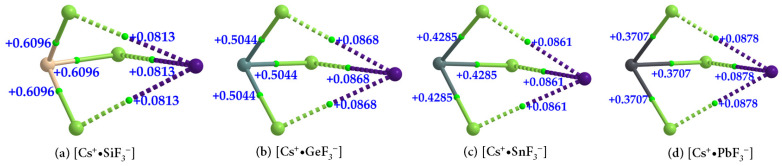
(**a**–**d**) QTAIM-based molecular graphs of cesium tetrel fluoride perovskite ion pairs, [Cs^+^•TtF_3_^−^] (Tt = Si, Ge, Sn, and Pb), obtained with [*ω*B97XD/def2-TZVPPD]. Values represent the Laplacian of the charge density (∇^2^ρ_b_/au) at the Tt–F and Cs···F bond critical points. Atoms, bond paths, and bond critical points are shown as large spheres, solid/dotted lines in atom color, and tiny spheres in green, respectively.

The formation of [Cs^+^•TtF_3_^−^] (Tt = Si, Ge, Sn, and Pb) ion pairs is also evident in the QTAIM-based molecular graphs shown in Figure 13a–d. In all cases, the ∇^2^*ρ*_b_ at the Tt–F bcps are positive, showing that they are closed-shell interactions and appreciably ionic. The values of ∇^2^*ρ*_b_ at Tt–F bcps across the series follow the order [Cs^+^•SiF_3_^−^] > [Cs^+^•GeF_3_^−^] > [Cs^+^•SnF_3_^−^] > [Cs^+^•PbF_3_^−^], which parallels the trend in the negative *H*_b_ values for the same bonds (Table 4); *H*_b_ < 0 indicates that the bonds possess some covalency. The character of these coordinate interactions deduced from ∇^2^*ρ*_b_ and *H*_b_ values are not the same as that found for the Cs···F bcps. For the latter, the sign of both ∇^2^*ρ*_b_ and *H*_b_ are positive (Table 4), indicative of closed-shell (non-covalent) interactions.

### 2.2. Oligomers of the [Cs^+^•PbI_3_^−^] Ion Pair

**Figure 14:**
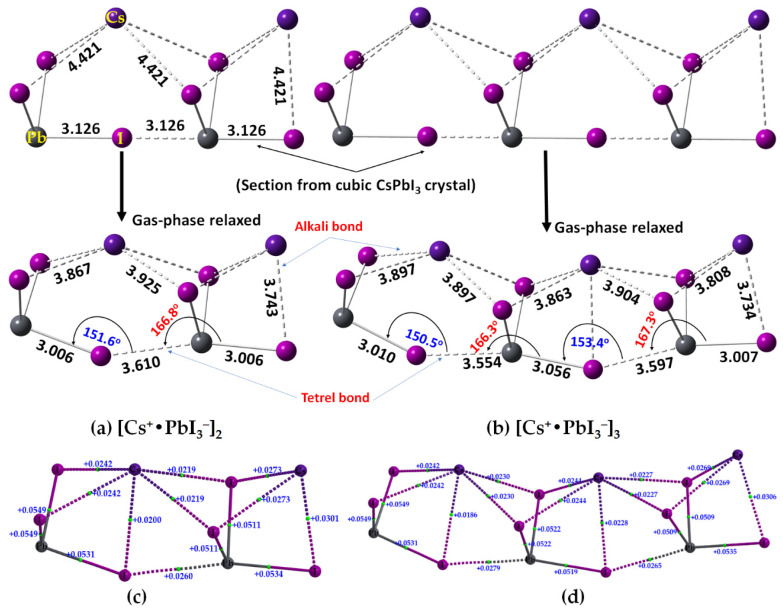
Illustration of sections from the cubic crystal of CsPbI_3_ (top) and the [*ω*B97XD/def2-TZVPPD]-level fully relaxed geometries (bottom) of (**a**) [Cs^+^•PbI_3_^−^]_2_ dimer and (**b**) [Cs^+^•PbI_3_^−^]_3_ trimer, showing selected bond distances and bond angles in Å and degree, respectively. Shown in (**c**,**d**) are the QTAIM molecular graphs, together with the Laplacian of the charge density at the bcps (a.u.) of the corresponding systems, respectively.

We have sectioned the supercell structure of cubic CsPbI_3_ (Figure 2a, bottom), and extracted the binary, trinary, and tertiary clusters in 1D. These were fully energy minimized at the same level of theory, [*ω*B97XD/def2-TZVPPD]. The geometries of the resulting [Cs^+^•PbI_3_^−^]_2_ dimer and [Cs^+^•PbI_3_^−^]_3_ trimer are shown in Figure 14a,b, respectively, together with their corresponding QTAIM-based molecular graphs in Figure 14c,d, respectively. The Pb–I bonds found in the crystal (top) are significantly elongated in the gas phase (bottom) (cf. Figure 14a,b). The ∠Pb–I···Pb angles between the ion pairs in cubic CsPbI_3_ are linear, but non-linear in the gas-phase structure, leading to significant deformation passing from the solid-state structure to the gas-phase dimer and trimer. This discrepancy between the gas-phase and the solid-state geometries is not very surprising given that the role of packing forces is absent in the former. Interestingly, both the gas phase structures resemble the tilting of edge-sharing Pb–I chains in 1D, observed in the case of 3D CsPbI_3_. The ∠Pb–I···Pb angles are between 150° and 154° (Figure 14a,b, bottom), close to that seen between the edge-shared [PbI_6_]^4−^ octahedra that are tilted relative to the corner-sharing octahedra in the low-temperature orthorhombic structure of CsPbI_3_ (∠Pb–I–Pb = 148.1° along the *a*-axis and 156.88° along the *c*-axis; ICSD ref: 17016 [49]). On the other hand, and as noted above, the tetrel bonds between the ion pairs are longer than the Pb–I coordinate bonds and are quasi-linear (∠I–Pb···I = 166.8° in [Cs^+^•PbI_3_^−^]_2_ (Figure 14a) and 166.3° and 167.3° in [Cs^+^•PbI_3_^−^]_3_ (Figure 14b). The physical chemistry of 1D CsPbI_3_ has been experimentally investigated [62,63,64]. It was shown that in the orthorhombic (*Pnma*) *γ*-phase, the PbI_6_^4−^ octahedra tilted around all three pseudocubic axes, *a^−^a^−^c^+^*, which is different to the tilt observed in the tetragonal (*P_4_/mbm*), *a^0^a^0^c^+^*, *β-*phase [65], and the bandgap increases with an increase in the octahedral tilting when the temperature cools down, allowing for the emergence of *β*-CsPbI_3_ and *γ*-CsPbI_3_ [66].

The results of our QTAIM analysis for the [Cs^+^•PbI_3_^−^]_2_ dimer (and [Cs^+^•PbI_3_^−^]_3_ trimer) are given in Table 5. The Pb–I coordinate bonds in the [Cs^+^•PbI_3_^−^]_2_ dimer (and [Cs^+^•PbI_3_^−^]_3_ trimer) are characterized by *ρ*_b_, ∇^2^*ρ*_b_ and −*H*_b_ values in the ranges of 0.0411–0.0472 (0.0379–0.0474), 0.0511–0.0116 (0.0509–0.0549), and 0.0071–0.007 (0.0058–0.0097) a.u., respectively. The corresponding values for the Cs···I alkali bonds were 0.0075–0.0116 (0.0069–0.0103), 0.0020–0.0301 (0.0186–0.0269), and −0.0008 (−0.0008) a.u., respectively. Although the former bonds possess a non-negligible amount of covalent character, the latter are purely electrostatic interactions. Their characteristics are comparable to those of I–Pb···I tetrel bonds (*ρ*_b_ = 0.0139 a.u., ∇^2^*ρ*_b_ = 0.0260 a.u., and −*H*_b_ = 0.0001 a.u). The [Cs^+^•PbI_3_^−^]_3_ trimer has two non-equivalent I–Pb···I tetrel bonds (3.554 and 3.597 Å, Figure 14b), with *ρ*_b_ = 0.0152 (0.0143) a.u., ∇^2^*ρ*_b_ = 0.0279 (0.0265) a.u., and *H*_b_ ≈ 0.00001 a.u for the shorter (longer) bonds. The detailed nature of ∇^2^*ρ*_b_ at various bcps is shown in Figure 14c,d for the [Cs^+^•PbI_3_^−^]_2_ dimer and [Cs^+^•PbI_3_^−^]_3_ trimer, respectively.

**Figure 15:**
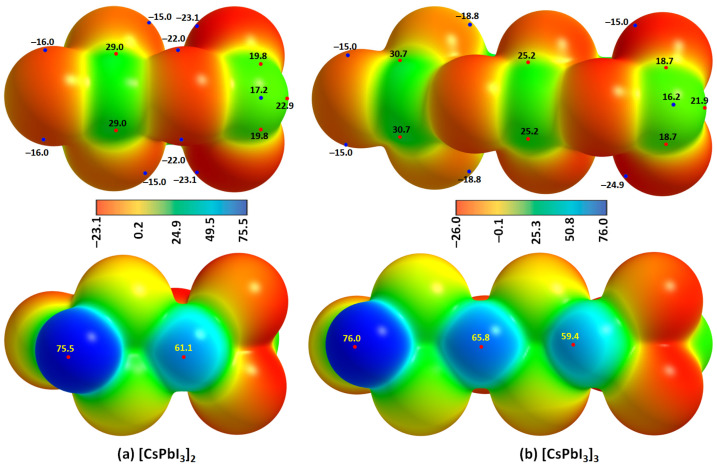
The [*ω*B97Xd/def2-TXVPPD] computed potential on the electrostatic surface of the (**a**) [Cs^+^•PbI_3_^−^]_2_ dimer and (**b**) [Cs^+^•PbI_3_^−^]_3_ trimer, mapped on their corresponding 0.001 a.u. isoelectronic density envelopes. The top and bottom panels represent that Pb and Cs sites of each Cs•PbI_3_ ion-pair in the dimer/trimer are facing the viewer, respectively. Selected local most maxima and minima of potential (*V_S,max_* and *V_S,min_*) represented by tiny circles in red and blue, respectively, are depicted. Values are given in kcal mol^−1^.

We confirmed the formation of I–Pb···I tetrel bonds in [Cs^+^•PbI_3_^−^]_2_ and [Cs^+^•PbI_3_^−^]_3_ oligomers using MESP results, Figure 15a,b. This signifies that one of the positive σ-holes on the surface of Pb in the [Cs^+^•PbI_3_^−^] ion pair (Figure 4a) is annihilated because it is engaged with the entirely negative (iodide) site in the neighboring ion pair through an electrostatic interaction, leading to the formation of an I–Pb···I tetrel bond. This causes a change in the potential minima and maxima on the surfaces of the two ion-pair entities at the equilibrium geometry of the oligomer.

An interesting feature of the [Cs^+^•PbI_3_^−^]_4_ tetramer is that the Pb–I bonds are no longer equivalent, as found in cubic CsPbI_3_ in 3D (Figure 4a), but comparable with those found in the [Cs^+^•PbI_3_^−^]_2_ dimer and [Cs^+^•PbI_3_^−^]_3_ trimer (*vide supra*). Again, this is the result of the gas phase, where the role of the periodic boundary condition is nullified and no packing forces act on the system. The [Cs^+^•PbI_3_^−^] ion pairs are free to interact with each other in the gas phase at 0 K, causing the linear Pb–I–Pb bonds found in the cubic structure of CsPbI_3_ to change appreciably in a manner so as to adopt a significantly distorted geometry very similar to that observed in the low-temperature orthorhombic phase of the system (*vide supra*).

**Figure 16:**
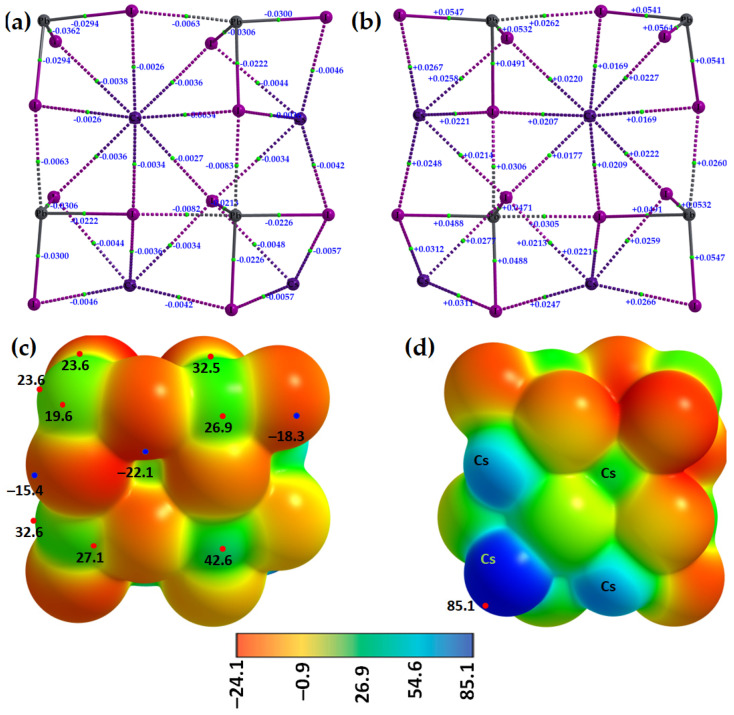
The QTAIM-based molecular graphs of the [Cs^+^•PbI_3_^−^]_4_ tetramer, showing the (**a**) potential energy density (*V*_b_) and (**b**) ∇^2^*ρ*_b_ values at various bcps. Atoms, bond paths, and bond critical points are shown as large spheres, solid/dotted lines in atom color, and tiny spheres in green, respectively. (**c**,**d**) represent two views of the same tetramer in which the Tt atoms in the former and the Cs atoms in the latter are facing the viewer. Selected local most maxima and minima of potential (*V_S,max_* and *V_S,min_*) represented by tiny circles in red and blue, respectively, are depicted in (**c**,**d**), with values shown in kcal mol^−1^. The atomic positions shown in the molecular graphs are the same as those in the MESP plots.

The molecular graphs of two different orientations of the [Cs^+^•PbI_3_^−^]_4_ tetramer are shown in Figure 16a (with values of the potential energy density, *V*_b_) and Figure 16b (∇^2^*ρ*_b_ at the bcps). Regardless of the nature of the bonding interactions involved, the sign of *V*_b_ is always negative and, hence, stabilizing. Values of ∇^2^*ρ*_b_ are all positive, indicating that the bonding interactions are of the closed-shell type. While the relationship *E*_b_(QTAIM) = −½ *V*_b_ may be empirical, it suggests that the I–Pb···I tetrel bond is stronger than the Cs···I alkali bond (*E*_b_(QTAIM) values of 4.0 kcal mol^−1^ vs. 1.5 kcal mol^−1^).

The formation of both Pb···I tetrel bonds and Cs···I alkali bonds between four units of the [Cs^+^•PbI_3_^−^] ion pairs in the [Cs^+^•PbI_3_^−^]_4_ tetramer can also be understood from the MESP plots shown in Figure 16c,d. Upon assembly, the σ-hole on three Pb atoms in three ion pairs of the tetramer is annihilated upon its attractive engagement with the iodide atom of a neighboring ion pair, thus forming Pb···I tetrel bonds. The four tetrel centers are positive (see the four green regions in Figure 16c), and one of them, which is not involved in the formation of the tetrel bond (Figure 16c, top left), conceives three σ-holes on its surface; these can accept nucleophiles when in close proximity to another three ion pairs. By contrast, the Cs ions are highly electrophilic. These unequivocally provide evidence of the fact that the formation of the 3D network of the cage-like structures of cesium tetrel halide perovskites are the result of σ-hole-centered tetrel-bonded interactions between [PbI_3_^−^] anions in the presence of Cs^+^. The physical chemistry of tetrel bonds also plays a significant role in stabilizing 1D CsPbI_3_, a material suitable for stable X-ray detection (sensitivity = 2.37 mC·Gy^−1^·cm^−2^, resistivity = 7.4 × 10^9^ Ω·cm, and carrier mobility–lifetime product = 3.63 × 10^−3^ cm^2^·V^−1^ [62]).

### 2.3. Stabilization Energy

The interaction energies and intermolecular bond distances between [Cs^+^] and [TtX_3_^−^] for all the sixteen [Cs^+^•TtX_3_^−^] ion pairs are summarized in Table 5. They are very large compared to ordinary non-covalent interactions but comparable with the binding energies of anion-molecule interactions. The charge-assisted tetrel bonds reported recently had energies (in kcal mol^−1^) as large as −93.43 [I_4_Ge···F^−^], –112.15 ([I_4_Si···F^−^]), and −84.05 ([I_4_Pb···F^−^]) with [CCSD/def2-TZVPPD], which were very close to those calculated with [*ω*B97X-D/def2-TZVPPD] [67]. Large interaction energies were also reported for tetrel bonds [62], halogen bonds [68], hydrogen bonds [68], and pnictogen bonds [69] in other anion–molecule complexes. The large interaction energies, *E*_b_ and *E*_b_(BSSE), summarized in Table 5 are expected since a large part of the contribution arises from coulombic interaction between two interacting charged moieties. The binding energies are also comparable with those reported for similar halide perovskite ion-pairs at the [CCSD(T)/cc-pVTZ] level of theory [70].

For a series with a given halogen derivative [Cs^+^•TtX_3_^−^], the interaction energies increase as the atomic size of the tetrel derivative increases (Si < Ge < Sn < Pb). This trend agrees well with the increasing strength of the σ-hole on Tt across the series for a given type of halogen derivative (see, for examples, Figure 4 for [Cs^+^•TtI_3_^−^], Figure 6 for [Cs^+^•TtBr_3_^−^], Figure 9 for [Cs^+^•TtCl_3_^−^], and Figure 12 for [Cs^+^•TtF_3_^−^]).

For a given tetrel derivative, the interaction energy increases as the atomic size of the halogen increases ([Cs^+^•TtF_3_^−^] > [Cs^+^•TtCl_3_^−^] > [Cs^+^•TtBr_3_^−^] > [Cs^+^•TtI_3_^−^]). For each series shown in Table 6, the largest interaction energy is calculated for cesium lead halide perovskite ion pairs, probably a consequence of the high polarizability of Pb compared to the other three tetrel derivatives. Among all the ion pairs examined, the ion pair of cesium lead fluoride perovskite, [Cs^+^•PbF_3_^−^], is the strongest. This is expected since fluorine in the inorganic moiety [PbF_3_^−^] is the most electronegative and electron-withdrawing of the halogens; hence, it strongly interacts with Cs^+^, which, therefore, cannot create strong σ-holes on Pb in [Cs^+^•PbF_3_^−^]. All these trends remain valid regardless of whether *E*_b_ or *E*_b_(BSSE) is considered since the BSSE is calculated to be very small.

**Figure 17:**
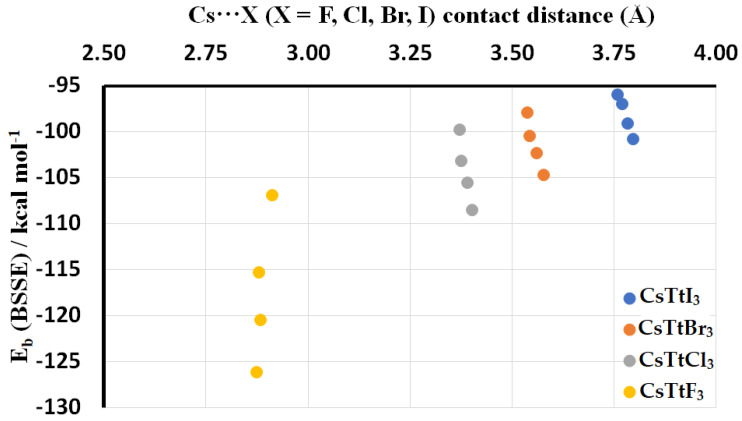
The relationship between interaction energy and the interionic distance in [Cs^+^•TtX_3_^−^] (Tt = Sn, Ge, Sn, Pb; X = F, Cl, Br, I) ion pairs.

As a rule of thumb, it is expected that the interaction energy increases as the intermolecular distance between interacting moieties decreases. However, this is not the case with the ion pairs explored in this study, where we find the opposite trend. The interaction energy for a given halogen derivative between [Cs^+^] and [TtX_3_^−^] increases when increasing the intermolecular distance between them (Table 6). This is not the case for ion pairs with a given tetrel derivative; here, the interaction energy increases ([Cs^+^•TtF_3_^−^] > [Cs^+^•TtCl_3_^−^] > [Cs^+^•TtBr_3_^−^] > [Cs^+^•TtI_3_^−^]) when decreasing the intermolecular bond distance ([Cs^+^•TtF_3_^−^] < [Cs^+^•TtCl_3_^−^] < [Cs^+^•TtBr_3_^−^] < [Cs^+^•TtI_3_^−^]), as seen in Table 5. The interaction energy associated with the alkali bonds as a function of bond distance for [Cs^+^•TtX_3_^−^] is shown in Figure 17.

The uncorrected binding energy of the tetrel bonds in the [Cs^+^•PbX_3_^−^]_2_ dimer, [Cs^+^•PbX_3_^−^]_3_ trimer, and [Cs^+^•PbX_3_^−^]_4_ tetramer shown in Figure 15 and Figure 16 are −20.7, −21.3, and −22.4 kcal mol^−1^, respectively. These were obtained by subtracting the total electronic energy of the oligomer from two, three, and four times the total electronic energy of the [Cs^+^•PbX_3_^−^] ion pair, indicating that the binding energy is nearly additive. However, these may not be solely due to the tetrel bonds since each Cs^+^ ion in each [Cs^+^•PbX_3_^−^] ion pair also contributes to the binding of the resulting oligomer through Cs···I alkali bonds. From these results, it is apparent that the empirical relationship *E*_b_(QTAIM) = −½*V*_b_ is not applicable to tetrel bond energies since it largely underestimates the magnitudes. The relationship might be useful for some hydrogen-bonded systems [71], but may not be generalized to other non-covalent interactions such as the tetrel bonds explored in this work.

## 3. Discussion

This study was undertaken to reveal the underlying reasons why the TtX_3_^−^ units interact to form the inorganic cage-like tetrel halide frameworks of 3D cesium halide perovskites. We have shown that the connectivity between the TtX_3_^−^ anions that lead to the formation of the 3D infinite inorganic framework, [TtX_3_^−^]_∞_, is driven by the inorganic cation, Cs^+^, through the effects of both electrostatic polarization and coulombic attraction. The joint involvement of electrostatic polarization and coulombic attraction causes redistribution of the charge density profile on the electrostatic surfaces of the molecular tetrel halide perovskites, resulting in the development of positive σ-holes on the Tt atom in the TtX_3_^−^ anions in the ion pairs [Cs^+^•TtX_3_^−^] (Tt = Ge, Sn, Pb). This is accompanied by appreciable amount of charge transfer from the halides of the TtX_3_^−^ anions to Cs^+^ when they are in close proximity. The σ-holes formed on the Tt atom, therefore, are able to simultaneously attract the negative halogens from three interacting TtX_3_^−^ anions, thus leading to the formation of TtX_6_^4−^ octahedra, the underlying framework of the 3D CsTtX_3_ cesium tetrel halide perovskites. The tetrel bonds formed are hidden between the anion moieties in the solid state structures, and to demonstrate their presence requires appropriate theoretical methods such as the QTAIM and MESP models.

There has been a failure to experimentally produce 3D tetrel halide perovskites when Tt = Si. We have explained this failure using the results of the MESP model. The underlying reason is the lack of an appreciable positive potential on the Si atom along the X–Si bond extensions in the molecular ion pair, [Cs^+^•SiX_3_^−^]. Therefore, Si is incapable of engaging with the negative halogen in a neighboring unit because of the columbic repulsion between them.

Although the fluorinated ion-pair systems displayed electrophilic regions when Tt = Pb and Sn, the latter does not form a perovskite structure because of the small size of the cavity formed by repeating [SnF_3_^−^] units, a cavity that cannot accommodate the inorganic cation. This might explain why CsSnF_3_ crystalizes in low-dimension. By contrast, the results of the MESP model have showed that CsGeF_3_ and CsSiF_3_ perovskites cannot be formed in the crystalline phase because the Tt along the F–Tt bond extensions in the [Cs^+^•SiF_3_^−^] (Tt = Ge, Si) ion pair has negative σ-holes. These negative σ-holes would repel the entirely negative fluorine atom(s) in a neighboring interacting unit(s), preventing the formation of Tt···F (Tt = Ge, Si) tetrel bonds. This also explains why the crystal structures of CsSiF_3_ (Tt = Ge, Si) are unknown.

## 4. Materials and Methods

### Computational Details

The geometries of sixteen ion pairs, [Cs^+^•TtX_3_^−^] (Tt = Pb, Sn, Ge, Si; X = I, Br, Cl, F), were fully energy-minimized, followed by frequency calculations. The most stable conformer was considered. The *ω*B97XD functional [72] as implemented in the Gaussian 16 code [73], together with the def2-TZVPPD basis set retrieved from the EMSL basis set library [74], was employed. *ω*B97XD is known as a range-separated functional and is capable of capturing both short-range and long-range interactions. Minenkov et al. have demonstrated that the *ω*B97XD functional outperforms other commonly used DFT functionals (PBE and TPSS, M06 and M06L) and also produces relatively small statistical errors when considering the overall structure and inter-nuclear distances [75]. All ion-pair geometries were at an energy minimum, confirmed by positive harmonic vibrational frequencies. Default convergence criteria (viz. tight SCF convergence and ultrafine integration grid) were invoked.

Similar calculations, as above, were also performed for a dimer, a trimer, and a tetramer of [Cs^+^•PbI_3_^−^], which we refer to as the [Cs^+^•PbI_3_^−^]*_n_* (*n* = 2, 3, 4) oligomers, to demonstrate the charge density topologies of alkali and tetrel bonding interactions responsible for the formation of the 3D network of CsPbI_3_. The nature of physical chemistry revealed for these systems might be transferable to other oligomers when Pb in [Cs^+^•PbI_3_^−^]*_n_* (*n* = 2, 3, 4) is replaced, for example, by Sn and Ge.

Relativistic spin-orbit coupling (SOC) is an important feature of tetrel halide perovskite semiconductors containing heavy atoms such as Pb because its inclusion affects the band structure without affecting the crystal geometry [76]. In particular, the inclusion of SOC can affect the direct character of the band gap transition between the extrema of valence and conduction band states, especially for periodic systems containing Pb [77]. This effect, called the Rashba–Dresselhaus effect [78], is observed specifically in non-centrosymmetric environments and can be directly measured by angle-resolved X-ray photoemission spectroscopy. However, because our calculations are aperiodic and do not involve the calculation of the band structures of the molecular entities considered, the effect of SOC was not taken into account, in line with several recent studies [67,79,80,81,82].

The uncorrected and BSSE-corrected interaction energies (*E*_b_ and *E*_b_(BSSE), respectively) of the ion pairs [Cs^+^•TtX_3_^−^] were calculated using Equations (1) and (2), respectively. BSSE refers to the basis set superposition error, evaluated using the counterpoise procedure of Boys and Bernardi [83], and *E*_T_ [Cs^+^•TtX_3_^−^], *E*_T_ [Cs^+^] and *E*_T_ [TtX_3_^−^] are the total electronic energies of the respective species.

*E*_b_ [Cs^+^•TtX_3_^−^] = *E*_T_ [Cs^+^•TtX_3_^−^] − *E*_T_ [Cs^+^] − *E*_T_ [TtX_3_^−^] (1)

*E*_b_ (BSSE) = *E*_b_ [Cs^+^•TtX_3_^−^] + BSSE (2)

QTAIM [84] calculations were performed at the same level of theory described above. Properties such as the charge density (*ρ*_b_), the Laplacian of the charge density (∇^2^*ρ*_b_), and the total energy density (*H*_b_) at the (3, −1) bond critical points (bcps), critical points where the gradients of *ρ*(***r***) vanish, were analyzed. The latter two properties at bcps provide insight into the closed-shell and/or open-shell nature of an interaction between a pair of atomic basins in molecular and intermolecular entities. For instance, the positive and negative signs of ∇^2^*ρ*_b_ were utilized to demonstrate the closed- and open-shell interactions, respectively, which were identified between the inorganic anion and cation and between the anions [85,86,87,88,89]. Similarly, the positive and negative signs of *H*_b_ (*H*_b_ = *G*_b_ + *V*_b_) were utilized to provide insight into the absence and presence of a covalent interaction between the corresponding moieties, respectively [86,90]. This relies on the fact that a positive *H*_b_ indicates a prevalence of the gradient kinetic energy density *G*_b_ over the potential energy density *V*_b_, which is typical of non-covalently bonded interactions [91,92,93]. We have also used the empirical formula *E*_b_(QTAIM) = −½ *V*_b_ [71] to calculate the binding energy associated with the various non-covalent interactions identified in the ion-pairs investigated.

The MESP calculations [94,95,96,97,98,99] were performed with [*ω*B97XD/def2-TZVPPD], utilizing the fully relaxed geometries of the ion pairs and oligomers. The signs and magnitudes of the potential extrema were computed using the 0.001 a.u. isoelectronic density envelope of the ion pairs. The magnitude of potential is a measure of the strength, whereas its (positive and negative) signs were used to provide insight into regions of charge density depletion and concentration on the electrostatic surfaces of the ion pairs, respectively. That is, th corresponding signs of the local most maxima and minima of potential (*V_S,max_* and *V_S,min_*, respectively) were utilized to arrive at these conclusions. For instance, the sign of both *V_S,max_* and *V_S,min_* can either be positive or negative, or sometimes even neutral. When positive, it is generally assumed that the region on the surface that accompanies this is electrophilic and, hence, may be suitable for accepting electron density from an interacting electron donor in close proximity. When it is negative, the region on the surface of the molecular entity that features this is nucleophilic and, hence, may be capable of donating electron density to an interacting electrophile when in close vicinity. However, it should be kept in mind that all negative or positive sites on the surface of the molecular entity may or may not always be capable of engaging in attractive interaction with a region that features the opposite reactive profile. 

An electrophilic σ-hole on atom A lying opposite to the R–A covalent bond is characterized when the sign *V_S,max_* is positive (*V_S,max_* > 0) [97,100]. Similarly, a nucleophilic σ-hole on atom A in R–A is observed when *V_S,max_* is negative (*V_S,max_* < 0) [97,100]. For instance, the σ-hole on F in H–F and H_3_C–F is negative, whereas that on X in H_3_C–X, X_3_C–X, and F_5_CX (X = Cl, Br, I) is positive. The underlying equation details and applicability of the MESP model to understand non-covalent interactions have appeared in several studies [67,95,96,99,101,102,103,104,105,106,107], hence we do not repeat them here.

AIMAll [108] and MultiWfn [109] codes were used for MESP and QTAIM analyses.

## Figures and Tables

**Table 1:** Selected QTAIM-based topological charge density properties at Tt–I and Cs···I bcps in [Cs^+^•TtI_3_^−^] (Tt = Si, Ge, Sn, Pb), obtained with [*ω*B97XD/def2-TZVPPD].

Species	Bond Type	*ρ*_b_/a.u.	∇^2^*ρ*_b_/a.u.	*H*_b_/a.u.
Cs^+^•SiI_3_^−^	Cs···I	0.0110	0.0292	0.0008
	Si–I	0.0629	−0.0515	−0.0293
Cs^+^•GeI_3_^−^	Cs···I	0.0104	0.0285	0.0009
	Ge–I	0.0570	0.0064	−0.0188
Cs^+^•SnI_3_^−^	Cs···I	0.0108	0.0284	0.0008
	Sn–I	0.0484	0.0386	−0.0119
Cs^+^•PbI_3_^−^	Cs···I	0.0104	0.0274	0.0008
	Pb–I	0.0451	0.0536	−0.0088

**Table 2:** Selected QTAIM-based topological charge density properties at Tt–Br and Cs···Br bcps of [Cs^+^•TtBr_3_^−^] (Tt = Si, Ge, Sn, Pb), obtained with [*ω*B97XD/def2-TZVPPD].

Species	Bond Type	*ρ*_b_/a.u.	∇^2^*ρ*_b_/a.u.	*H*_b_/a.u.
Cs^+^•SiBr_3_^−^	Cs···Br	0.0122	0.0377	0.0011
	Si–Br	0.0689	−0.0310	−0.0371
Cs^+^•GeBr_3_^−^	Cs···Br	0.0122	0.0374	0.0011
	Ge–Br	0.0662	0.0314	−0.0245
Cs^+^•SnBr_3_^−^	Cs···Br	0.0119	0.0363	0.0011
	Sn–Br	0.0547	0.0811	−0.0121
Cs^+^•PbBr_3_^−^	Cs···Br	0.0116	0.0352	0.0011
	Pb–Br	0.0514	0.0918	−0.0092

**Table 3:** Selected QTAIM-based topological charge density properties at Tt–Cl and Cs···Cl bcps of [Cs^+^•TtCl_3_^−^] (Tt = Si, Ge, Sn, Pb) ion pairs, obtained with [*ω*B97XD/def2-TZVPPD].

Species	Bond Type	*ρ*_b_/a.u.	∇^2^*ρ*_b_/a.u.	*H*_b_/a.u.
Cs^+^•SiCl_3_^−^	Cs···Cl	0.0136	0.0472	0.0017
	Si–Cl	0.0754	0.0502	−0.0400
Cs^+^•GeCl_3_^−^	Cs···Cl	0.0136	0.0468	0.0016
	Ge–Cl	0.0746	0.0778	−0.0287
Cs^+^•SnCl_3_^−^	Cs···Cl	0.0133	0.0455	0.0016
	Sn–Cl	0.0621	0.1268	−0.0147
Cs^+^•PbCl_3_^−^	Cs···Cl	0.0131	0.0445	0.0016
	Pb–Cl	0.0581	0.1311	−0.0111

**Table 4:** Selected QTAIM-based topological charge density properties at Tt–F and Cs···F bcps of [Cs^+^•TtF_3_^−^] (Tt = Si, Ge, Sn, Pb) ion pairs, obtained with [*ω*B97XD/def2-TZVPPD].

Species	Bond Type	*ρ*_b_/a.u.	∇^2^*ρ*_b_/a.u.	*H*_b_/a.u.
Cs^+^•SiF_3_^−^	Cs···F	0.0181	0.0813	0.0028
	Si–F	0.1060	0.6096	−0.0327
Cs^+^•GeF_3_^−^	Cs···F	0.0196	0.0868	0.0028
	Ge–F	0.1070	0.5044	−0.0316
Cs^+^•SnF_3_^−^	Cs···F	0.0197	0.0861	0.0026
	Sn–F	0.0911	0.4285	−0.0209
Cs^+^•PbF_3_^−^	Cs···F	0.0202	0.0878	0.0026
	Pb–F	0.0828	0.3707	−0.0152

**Table 5:** Selected QTAIM-based charge density properties at Pb–I, Cs···I, and Pb···I bcps of [Cs^+^•PbI_3_^−^]_2_ and [Cs^+^•PbI_3_^−^]_3_ oligomers, obtained with [*ω*B97XD/def2-TZVPPD].

Property	[Cs^+^•PbI_3_^−^]_2_	[Cs^+^•PbI_3_^−^]_3_
	Pb–I coordinate bond	
***ρ*** **_b_** **/a.u.**	0.0411–0.0472	0.0379–0.0474
**∇** **^2^** ***ρ*** **_b_** **/a.u.**	0.0511–0.0116	0.0509–0.0549
***H*_b_** **/a.u.**	−(0.0071–0.0072)	−(0.0058–0.0097)
	Cs···I alkali bond	
***ρ*** **_b_** **/a.u.**	0.0075–0.0116	0.0069–0.0103
**∇** **^2^** ***ρ*** **_b_** **/a.u.**	0.0020–0.0301	0.0186–0.0269
***H*_b_** **/a.u.**	0.00078	0.00078
	Pb···I tetrel bond	
***ρ*** **_b_** **/a.u.**	0.0139	0.0152 (0.0143) ^a^
**∇** **^2^** ***ρ*** **_b_** **/a.u.**	0.0260	0.0279 (0.0265) ^a^
***H*_b_** **/a.u.**	0.00013	−0.000083 (0.000018) ^a^

^a^ Properties correspond to two non-equivalent tetrel bonds (see text for discussion).

**Table 6:** The [*ω*B97Xd/def2-TXVPPD]-level uncorrected and BSSE-corrected binding energies (*E*_b_ and *E*_b_(BSSE), respectively) and alkali and coordinate bond distances (*r*(Cs···X) and *r*(Tt–X), respectively) of [Cs^+^•TtX_3_^−^] (Tt = Si, Ge, Sn, Pb; X = F, Cl, Br, I) ion pairs.

Ion Pair	*E*_b_ (kcal mol^−1^)	*E*_b_(BSSE) (kcal mol^−1^)	*r*(Cs···X) (Å)	*r*(Tt–X) (Å)
[Cs^+^•SiI_3_^−^]	−96.07	−96.05	3.759	2.635
[Cs^+^•GeI_3_^−^]	−97.10	−97.02	3.770	2.723
[Cs^+^•SnI_3_^−^]	−99.13	−99.11	3.785	2.889
[Cs^+^•PbI_3_^−^]	−100.89	−100.87	3.798	2.969
[Cs^+^•SiBr_3_^−^]	−98.13	−98.00	3.539	2.403
[Cs^+^•GeBr_3_^−^]	−100.66	−100.50	3.544	2.500
[Cs^+^•SnBr_3_^−^]	−102.51	−102.38	3.561	2.668
[Cs^+^•PbBr_3_^−^]	−104.84	−104.72	3.578	2.754
[Cs^+^•SiCl_3_^−^]	−99.92	−99.81	3.373	2.226
[Cs^+^•GeCl_3_^−^]	−103.37	−103.24	3.377	2.335
[Cs^+^•SnCl_3_^−^]	−105.74	−105.63	3.392	2.506
[Cs^+^•PbCl_3_^−^]	−108.68	−108.58	3.403	2.600
[Cs^+^•SiF_3_^−^]	−106.99	−106.91	2.914	1.706
[Cs^+^•GeF_3_^−^]	−115.46	−115.37	2.881	1.852
[Cs^+^•SnF_3_^−^]	−120.61	−120.54	2.885	2.033
[Cs^+^•PbF_3_^−^]	−126.32	−126.24	2.876	2.144

## Data Availability

This research did not report any data.
